# Learning cortical hierarchies with temporal Hebbian updates

**DOI:** 10.3389/fncom.2023.1136010

**Published:** 2023-05-24

**Authors:** Pau Vilimelis Aceituno, Matilde Tristany Farinha, Reinhard Loidl, Benjamin F. Grewe

**Affiliations:** ^1^Institute of Neuroinformatics, University of Zurich and ETH Zurich, Zurich, Switzerland; ^2^ETH AI Center, ETH Zurich, Zurich, Switzerland

**Keywords:** cortical hierarchies, deep learning, credit assignment, synaptic plasticity, backpropagation, spiking time-dependent plasticity, target propagation, differential Hebbian learning

## Abstract

A key driver of mammalian intelligence is the ability to represent incoming sensory information across multiple abstraction levels. For example, in the visual ventral stream, incoming signals are first represented as low-level edge filters and then transformed into high-level object representations. Similar hierarchical structures routinely emerge in artificial neural networks (ANNs) trained for object recognition tasks, suggesting that similar structures may underlie biological neural networks. However, the classical ANN training algorithm, backpropagation, is considered biologically implausible, and thus alternative biologically plausible training methods have been developed such as Equilibrium Propagation, Deep Feedback Control, Supervised Predictive Coding, and Dendritic Error Backpropagation. Several of those models propose that local errors are calculated for each neuron by comparing apical and somatic activities. Notwithstanding, from a neuroscience perspective, it is not clear how a neuron could compare compartmental signals. Here, we propose a solution to this problem in that we let the apical feedback signal change the postsynaptic firing rate and combine this with a differential Hebbian update, a rate-based version of classical spiking time-dependent plasticity (STDP). We prove that weight updates of this form minimize two alternative loss functions that we prove to be equivalent to the error-based losses used in machine learning: the inference latency and the amount of top-down feedback necessary. Moreover, we show that the use of differential Hebbian updates works similarly well in other feedback-based deep learning frameworks such as Predictive Coding or Equilibrium Propagation. Finally, our work removes a key requirement of biologically plausible models for deep learning and proposes a learning mechanism that would explain how temporal Hebbian learning rules can implement supervised hierarchical learning.

## 1. Introduction

To survive in complex natural environments, humans and animals transform sensory input into neuronal signals which in turn generate and modulate behavior. Learning of such transformations often amounts to a non-trivial problem, since sensory inputs can be very high-dimensional and complex. The complexity of sensory inputs requires hierarchical information processing, which relies on multilayer networks. To form hierarchies, cortical networks need to process these sensory signals and convey plasticity signals down to every neuron in the hierarchy so that the output of the network (e.g., the motor output or behavior) improves during learning. In deep learning, this is known as the credit assignment (CA) problem and it is commonly addressed by the error backpropagation (BP) method. During BP learning, neurons in the lower hierarchies change their afferent synapses by integrating a backpropagated error signal. A neuron's afferent weight update is then calculated as the product of the presynaptic activity and its non-local output error. However, several key aspects of BP are still at odds with learning in biological neural networks (Crick, 1989; Lillicrap et al., 2020). For example, ANNs separate the processing or encoding of neuronal activity signals from the weight update signals, they utilize distinct phases and they implement an exact weight symmetry of forward and feedback pathways. Moreover, plasticity in biological synapses is local in space and time and tightly coupled to the timing of the pre- and post-synaptic activity (Bi and Poo, 1998).

**Figure 1:**
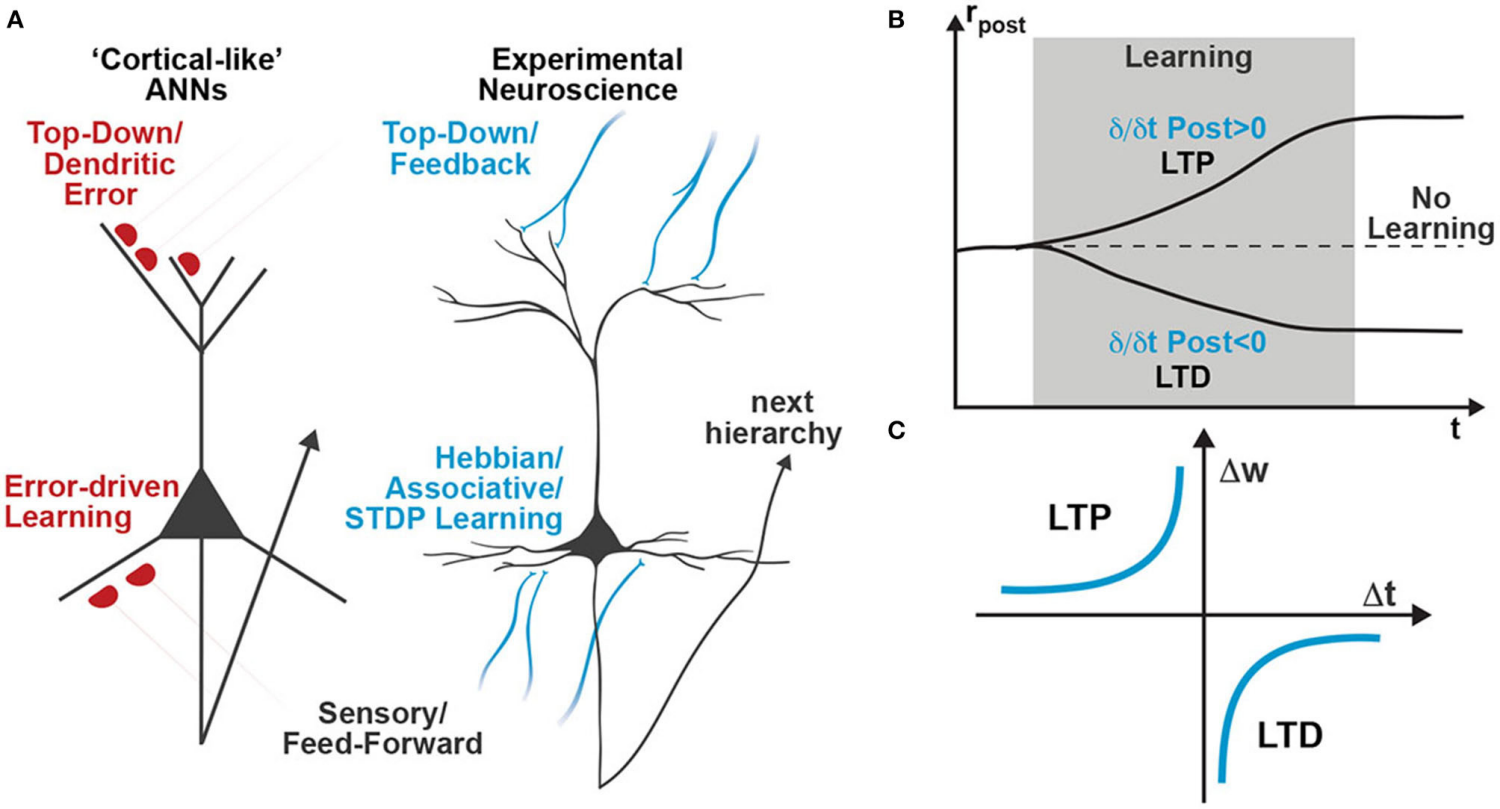
Schematic comparison of learning rules in artificial and biological neural networks. **(A)** While recently proposed cortical-like ANNs utilize dendritic-error learning rules to induce plasticity in basal synapses (left neuron), biologically observed plasticity rules are based on Hebbian-type associative learning rules such as STDP (right neuron). **(B)** A temporal Hebbian update rule such as STDP directly relates to increasing or decreasing postsynaptic activity. Thus, STDP learning is also often referred to as differential Hebbian learning (Xie and Seung, 1999; Zappacosta et al., 2018). **(C)** Classical STDP profile showing ranges of Δ t that induce long-term potentiation (LTP) and long-term depression (LTD), as extracted from experimental observations in neuroscience.

Attempting to address some of these implausibilities, recent cortical-inspired ANN models leverage network dynamics to directly couple changes in neuronal activity to weight updates (Whittington and Bogacz, 2017; Sacramento et al., 2018). Those models postulate multi-compartment pyramidal neurons with a highly specialized dendritic morphology that use their apical dendrite to integrate a feedback signal that modulates feedforward plasticity (Figure 1A, left schematic). Although multi-compartment models agree with some biological constraints, such as the spatial locality of learning rules and the fact that feedback not only generates plasticity but also affects neuronal activity (Gilbert and Li, 2013), the apical “dendritic-error” learning approach still requires tightly coordinated and highly specific error signaling circuits (Whittington and Bogacz, 2017; Sacramento et al., 2018). To avoid these highly specific error circuits, we recently developed a novel class of cortical-inspired ANNs that utilizes the same dendritic-error learning rule, but does not require highly specific error circuits and is capable of online learning of all weights without requiring separate forward and backward passes (Meulemans et al., 2021a,b, 2022b). In this model, known as “Deep Feedback Control” (DFC), we dynamically tailor the feedback to each hidden neuron until the network output reaches the desired target. The weight update of the feedforward pathway is then calculated upon convergence as the difference in neural activities when the effect of top-down apical feedback is fully taken into account or not. Still, this model relies on the same dendritic-error learning rule as its predecessors (Whittington and Bogacz, 2017; Sacramento et al., 2018), and it is unclear how a neuron would be able to compare the activities of its basal and apical compartments (Figure 1A, left scheme). In this work, we argue that dendritic learning rules can be substituted by experimentally validated temporal Hebbian learning rules (e.g., STDP) and we use the DFC framework as an example of how a deep network can learn with this mechanism. We argue that single-compartment neurons, whose firing rate is strongly affected by apical input, can use the difference between consecutive instances of their activity as a learning signal (Figure 1B), as opposed to comparing the changes in two different compartments. Based on this dynamic change in the postsynaptic activity we can thus encode the learning signal while being consistent with experimentally observed learning rules such as STDP (Figure 1C).

Box 1 **Spike-Timing Dependent Plasticity (STDP)**

When using the term STDP, we here refer to the well-established observation that the precise timing of pre- and post-synaptic spikes significantly determines the sign and magnitude of synaptic plasticity (Markram et al., 1997; Bi and Poo, 1998). In cortical pyramidal neurons, a presynaptic spike that precedes a postsynaptic spike within a narrow time window induces long-term potentiation (LTP) (Markram et al., 1997; Bi and Poo, 1998; Nishiyama et al., 2000; Sjöström et al., 2001; Wittenberg and Wang, 2006; Feldman, 2012); if the order is reversed it leads to long-term depression (LTD). Using this classical STDP profile (Figure 1A), multiple theoretical models were able to predict biological plasticity by assuming a simple superposition of spike pairs (Gerstner et al., 1996; Kempter et al., 1999; Abbott and Nelson, 2000; Song et al., 2000; van Rossum et al., 2000; Izhikevich and Desai, 2003; Gütig, 2016).

## 2. Results

### 2.1. Single neuron supervised learning with STDP

**Figure 2:**
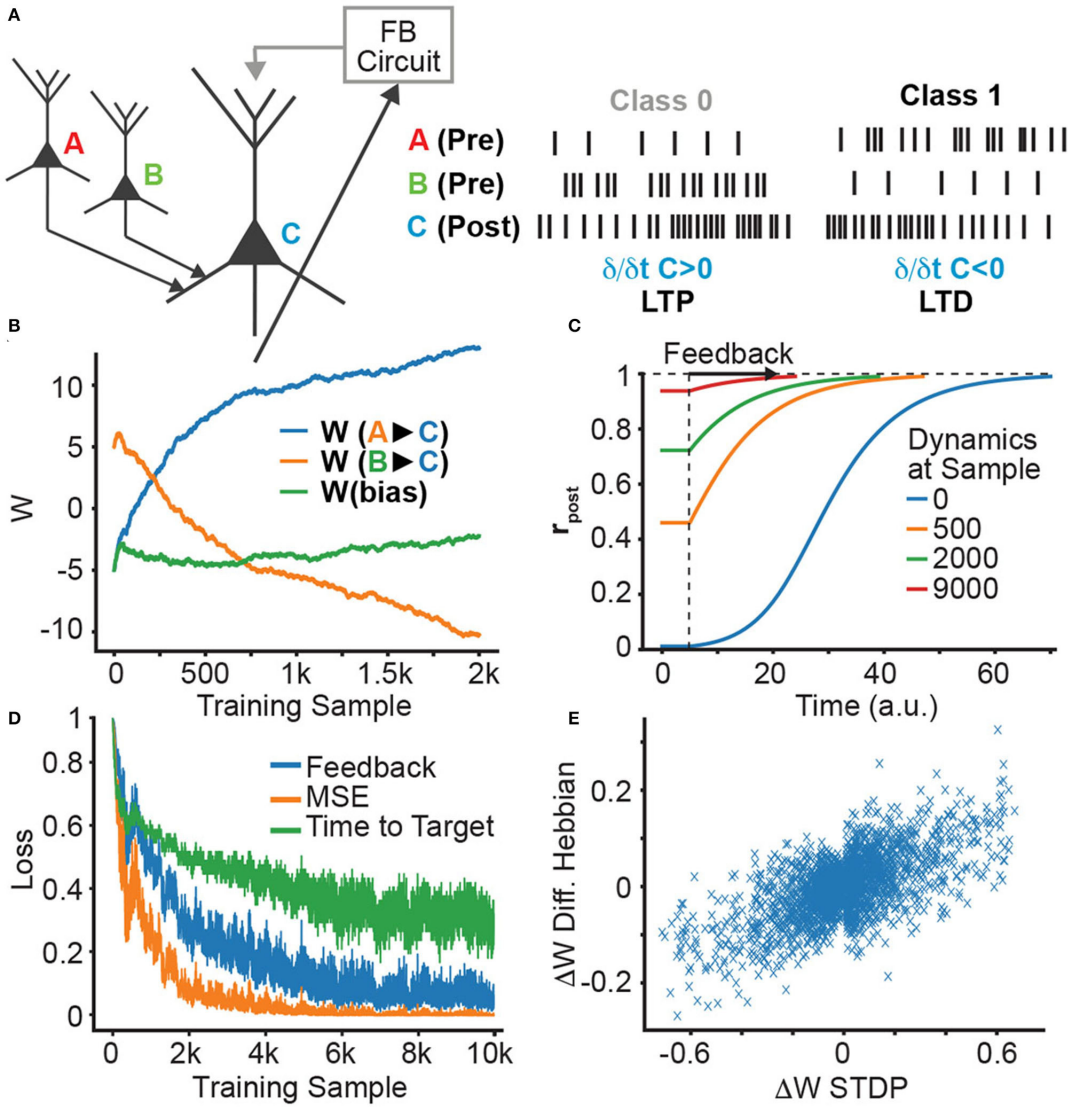
Single neuron supervision and STDP learning. **(A)** Single neuron supervision scheme. Throughout learning, we plot: **(B)** the evolution of the presynaptic weights originating from neurons A and B; **(C)** the evolution of the neural dynamics; **(D)** the decreasing feedback, MSE loss, and time-to-target; and **(E)** the correlation between DH and STDP weight updates.

We first demonstrate how an STDP learning rule can be used to train a single neuron on a linear classification task (Figure 2A). We use a neuron with the sigmoid activation function, which gets both feedforward basal inputs from two other neurons (A and B) and a feedback apical input, resulting in the rate-based dynamics


(1) $$\begin{array}{l} {\dot{v}}_{\text{post}}(t)=-v_{\text{post}}(t)+w_{A}r_{A}+w_{B}r_{B}+c(t)\\ r_{\text{post}}(t)=\phi \left(v_{\text{post}}(t)\right) \end{array}$$

where *w*_*A*_, *w*_*B*_ are the synaptic strengths of the connection from the input neurons to the output neuron, *r*_*A*_, *r*_*B*_ are the firing rates of neurons *A, B*, respectively, *r*_post_(*t*) and *v*_post_(*t*) are the output firing rate and membrane potential at time *t*, and *c*(*t*) is the apical feedback given to the output neuron. In our simple example, the neuron can get two incoming stimuli, from neurons *A* and *B*, and the apical feedback *c*(*t*) changes the output firing rate to be high when *B* is presented and low when *A* is presented.

The firing rate variables are converted into spike trains with an inhomogeneous Poisson process, where at every time step the probability of spiking in each neuron is given by *r*_post_(*t*), *r*_*A*_, *r*_*B*_, respectively. These spike trains are then used to induce synaptic weight changes by STDP (see Figure 2B).

We observe in Figure 2B that, as learning progresses, the weights evolve to the expected values (high for *w*_*B*_, low for *w*_*A*_), and that this changes the dynamics of *r*_post_(*t*), causing *r*_post_ to start closer to its target value and, thus, shortening the time and the feedback required to produce the desired output (Figures 2C, D). Such changes can be understood in terms of the following set of equivalent loss functions that are minimized:

- The initial distance to the target activity can be computed as the Mean Squared Error (MSE), denoted by L. This loss is commonly used in the machine learning literature as a standard performance measure.
- The feedback required to maintain or reach the target activity, denoted by H. This loss is equivalent to the one presented in previous works on using feedback to train neural networks (Gilra and Gerstner, 2017; Meulemans et al., 2020) and relates to the intuition from Predictive Coding that a trained ANN minimizes the feedback needed to correctly process the input (Rao and Ballard, 1999).
- The time delay to reach the target is denoted by T. This loss function represents the amount of time a neuron takes to reach its target value. This idea appears in previous works based on STDP models (Masquelier et al., 2009; Vilimelis Aceituno et al., 2020) and is also implicitly used in models for learning in deep networks (Luczak et al., 2022).

To relate the three losses to temporal Hebbian learning, we re-express the STDP update through its rate-based form, known as the differential Hebbian (DH) learning rule (Xie and Seung, 1999; Saudargiene et al., 2004; Bengio et al., 2017),


(2) $$\Delta w\propto \int r_{\text{pre}}(t){\dot{r}}_{\text{post}}(t)dt,$$

where Δ*w* is the change in feedforward synaptic strength, *r*_pre_(*t*) is the presynaptic activity and ṙ_post_(*t*) is the derivative of the postsynaptic activity, which corresponds to the change in firing probability. As we see in Figure 2E, the DH learning rule is indeed similar to STDP, albeit with noise induced by the inherent stochasticity of the Poisson neuron.

To understand how this rule relates to the three loss functions mentioned above, we note that in the single neuron example, the presynaptic firing rate is fixed, which simplifies the previous rule to


(3) $$\Delta w\propto r_{\text{pre}}\int {\dot{r}}_{\text{post}}(t)dt=r_{\text{pre}}\left[r_{\text{post}}(T)-r_{\text{post}}(0)\right],$$

where *r*_post_(*T*) is the postsynaptic activity after reaching the target state. This corresponds to the dendritic-error learning rule (Gilra and Gerstner, 2017; Sacramento et al., 2018; Meulemans et al., 2020). The correlation of the weight updates for this rule and STDP is shown in Figure 2E. In this single neuron setting, it is clear that both the STDP and DH learning mechanisms decrease the three loss functions: having an initial activity that is closer to the target activity implies that the MSE loss is lower at the beginning, and also that the change in activity is smaller. Hence, it needs less feedback and the target can be reached much faster (see Appendix, Section 2 for a detailed explanation).

The next key question is whether we can use STDP and DH learning in a similar manner for hierarchical credit assignment, i.e., for training multilayer neural networks.

### 2.2. Differential Hebbian can train multilayer networks

To extend our results to multilayer neural networks, the feedback must be received by the neurons in all layers. To compute the appropriate feedback signals, we use the framework of deep feedback control (DFC) from our previous work (Meulemans et al., 2021a), which we detail here for completeness.

**Figure 3:**
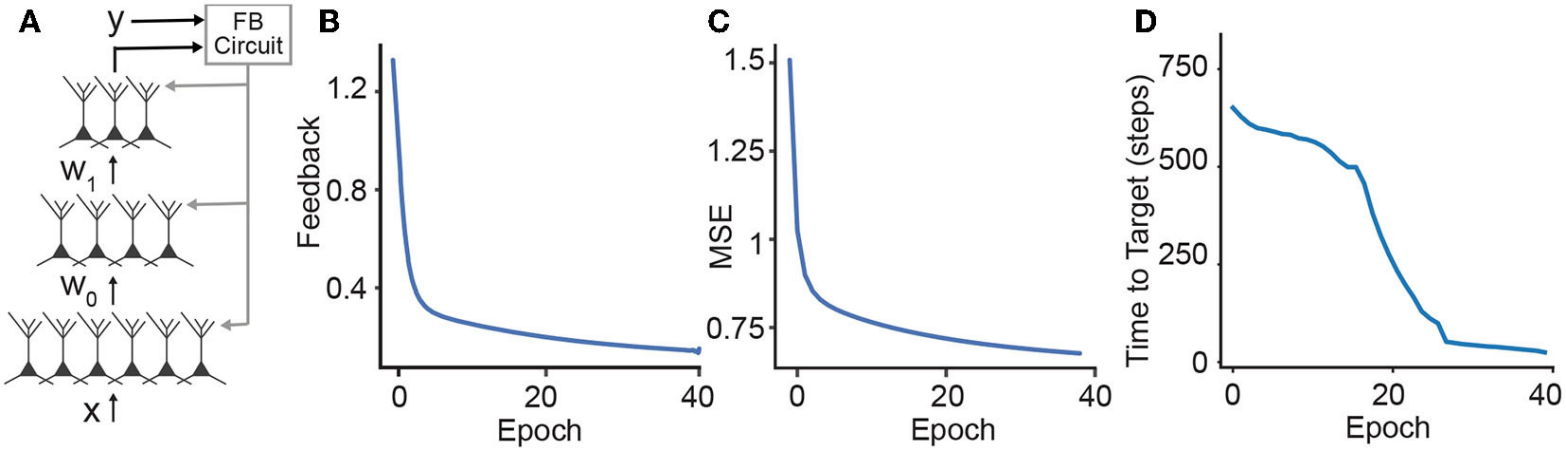
Learning in hierarchical networks. **(A)** Schematic illustration of how the feedback can be used to train a deep network. We use the desired output label and the output of the network to compute the feedback signals that are sent to the apical dendrites of the neurons. **(B–D)** Throughout the training, the three losses (feedback, MSE loss, and time-to-target) decrease for the MNIST benchmark.

In DFC, each neuron receives a feedforward basal input and an apical feedback signal that is computed by a controller whose goal is to achieve a target output response (Figure 3A). The neuronal dynamics is described by


(4) $${\dot{v}}_{\text{post}}(t)=-v_{\text{post}}(t)+\underset{\text{feedforward}}{\underset{︸}{W_{\left\{\text{pre},\text{post}\right\}}r_{\text{pre}}(t)}}+\underset{\text{feedback}}{\underset{︸}{Q_{\text{post}}c(t)}},$$

where the postsynaptic membrane potential **v**_post_(*t*) at each neuron is given by the presynaptic firing rate **r**_pre_(*t*), originating from neurons in the previous layer, multiplied by the feedforward synaptic weights *W*_{pre, post}_. In order to compute the target signal for every neuron, DFC uses a global PI controller that affects all the neurons in the network denoted by **c**(*t*),


(5) $$c(t)=c^{\text{int}}(t)+ke(t),\;\tau_{u}{\dot{c}}^{\text{int}}(t)=e(t)\alpha -c^{\text{int}}(t),$$

where *k* is the proportional control constant, **e**(*t*) is the difference between the target output activity and the network's current output, and α is the leak constant. The *Q* matrix contains the top-down feedback weights that map the controller signal into each hidden neuron and is pre-trained using local anti-Hebbian learning rules, as done in Meulemans et al. (2021a), but then kept fixed throughout the learning of the feedforward weights. We start with a random *Q* weight matrix and add independent zero-mean noise into the network,


(6) $${\dot{v}}_{\text{post}}(t)=-v_{\text{post}}(t)+W_{\left\{\text{pre},\text{post}\right\}}r_{\text{pre}}(t)+Q_{\text{post}}c(t)+\epsilon ,$$

where the fluctuations (ϵ) on every neuron propagate through the feedforward network and affect the output layer, which in turn creates fluctuations in **c**(*t*) that the controller then acts to eliminate them. We then use an anti-Hebbian learning rule of the form


(7) $${\dot{Q}}_{\text{post}}(t)=-v_{\text{post}}(t)c{\left(t\right)}^{T}-\beta Q_{\text{post}},$$

where parameter β controls the strength of the feedback weights. As proven in Meulemans et al. (2021a), learning *Q* with this rule ensures that the model does principled optimization, meaning it converges in learning.

We test the DH learning with the DFC setting on MNIST (LeCun, 1998), a widely accepted standard computer vision benchmark that aims to classify 28 × 28 pixel grayscale images of handwritten digits between 0 and 9. We show that DH with feedback computed through the DFC framework can train a three-hidden layer network (256 × 256 × 256) to match state-of-the-art performances and compare our framework with BP as well as the original DFC framework based on dendritic-error learning (Table 1). We find that the testing classification error rates of BP, DFC, and DH-DFC are on par with this benchmark.

**Table 1:** MNIST classification error for BP, DFC, DH-DFC, PC, and DH-PC.

	**MNIST (%)**
BP	1.74^±0.10^
DFC	1.98^±0.05^
DH-DFC	1.89^±0.15^
PC^*^	2.71^±0.2^
DH-PC^*^	2.69^±0.15^

We compare the testing accuracies reached by our training procedure (DH-DFC) with those achieved by BP, DFC (reported in Meulemans et al., 2021b), PC, and PC with DH (DH-PC) in the MNIST dataset. The reported values correspond to the average testing classification error obtained from five different random seeds. ^*^The PC and DH-PC are based on the code from Tschantz (2020) without any fine-tuning.

To complement our analysis, we investigate the training loss in DH-DFC. We note that the amount of feedback required to reach the target decreases throughout the training (Figure 3B), implying that DH-DFC also decreases the required feedback. The MSE loss also decreases (Figure 3C), hence DH-DFC also learns by minimizing an implicit error. Finally, we show that the latency to reach the target is also reduced (see Figure 3D), implying that the latency-reduction nature of temporally asymmetric learning rules (Masquelier et al., 2009; Vilimelis Aceituno et al., 2020) is reflected in our framework.

**Figure 4:**
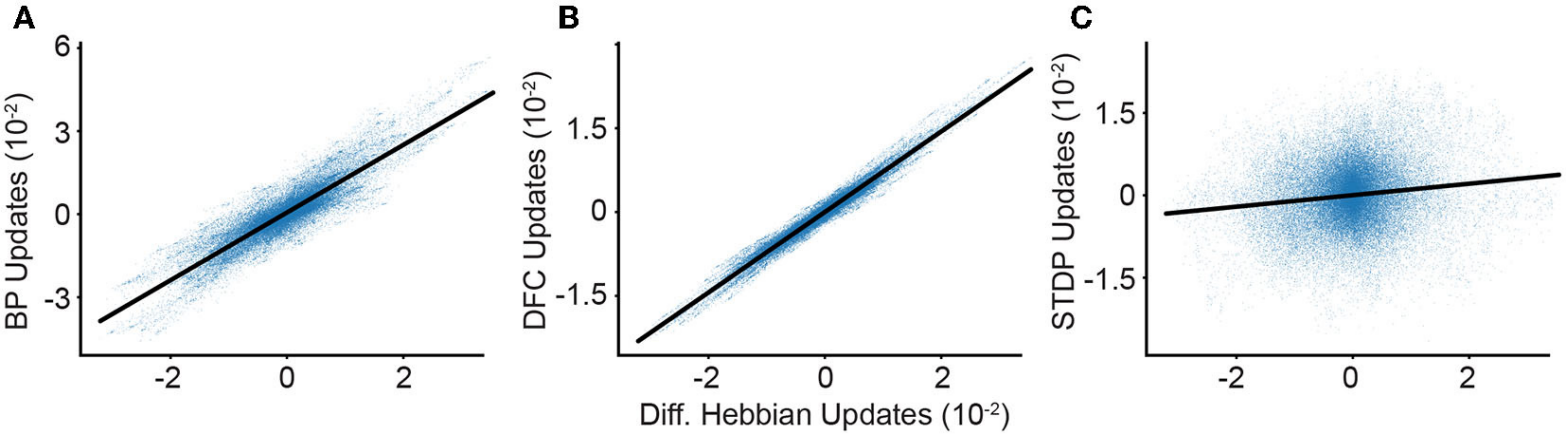
Comparison of learning algorithms. We calculate the synaptic weight update in our deep network using different algorithms and synaptic plasticity rules. We compare our DH weight updates (x-axis) to the updates given by other algorithms. Weight update correlations between DH-DFC and: **(A)** BP; **(B)** DFC; **(C)** and STDP. We observe a clear correlation between DH-DFC and both BP and DFC, and a significant but weaker correlation with STDP.

In addition, we experimentally calculate the similarity of the weight updates arising from different learning rules (Figure 4). We find that both the DFC and the BP updates are strongly positively correlated with DH-DFC, with coefficients of determination of 0.804 and 0.966, respectively.

Finally, we compare the DH-DFC weight updates with STDP updates evaluated on spike trains, which have a positive correlation, with a coefficient of determination of 0.008, but a very noisy alignment due to the randomness induced by using Poisson neurons (see Appendix, Section 5). This randomness can be reduced by computing several parallel conversions of firing rates to Poisson spike trains and averaging the resulting STDP updates, although here we find that limitations in computer memory prevent us from reaching state-of-the-art accuracies (see Appendix, Section 5).

It is worth noting that the DFC framework we use as a baseline is not the only model that uses feedback to train deep neural networks. In the next section, we argue that the use of temporal Hebbian rules is not restricted to the DFC framework, being instead applicable to other feedback-based learning models.

### 2.3. Differential Hebbian learning applies to other feedback-based networks

We extend our results on the single neuron framework and DFC multilayer model and prove that DH learning works in a general framework where some feedback is given to each neuron in the network so that the neuron reaches its target state. In contrast to the single neuron set-up, the DH learning rule is not equivalent to a simple delta rule. Since the presynaptic firing rate of most synapses changes in time, the DH learning rule can then be expressed as


(8) $$\Delta w\propto \int r_{\text{pre}}(t){\dot{r}}_{\text{post}}(t)dt=r_{\text{pre}}(T)\int {\dot{r}}_{\text{post}}(t)dt-\int {\tilde{r}}_{\text{pre}}(t){\dot{r}}_{\text{post}}(t)dt,$$

where the extra term includes the difference between the presynaptic firing rate at time *t* and its target, ${\tilde{r}}_{\text{pre}}\left(t\right)=r_{\text{pre}}\left(T\right)-r_{\text{pre}}\left(t\right)$.

To understand why DH learning works despite being different from the classical dendritic-error learning rule, it is useful to note that ${\tilde{r}}_{\text{pre}}\left(t\right)\to 0$ as learning proceeds, and, thus, this term disappears around the convergence point of the weights. By using an inductive argument, this can be extended to other layers (see Appendix, Section 3.1 for a detailed derivation).

A critical point of our convergence proof is that it does not depend on how feedback is computed. In fact, the only requirements are that feedback somehow pushes the neurons toward their target states. This suggests that the logic of DH learning should also work with other feedback-based learning models such as the original DFC (Meulemans et al., 2022a), but also models relying on Predictive Coding (PC) (Whittington and Bogacz, 2017; Rosenbaum, 2022), or Equilibrium Propagation (Scellier and Bengio, 2017). For DFC, we already saw that the weight updates align with DFC-DH and the performances are equally comparable (Figure 4 and Table 1); we further complement this by analytically showing that the learning rules converge to the same network configurations after learning (see Appendix, Section 4.1). For PC, we find that using the prediction error (implemented through error neurons) as implicit feedback leads to the same convergence proof as in the DH-DFC (see Appendix, Section 4.2). Moreover, in simulations, we find that the performance of PC using DH (DH-PC) is similar to that of PC and DH-DFC (see Table 1). For Equilibrium Propagation (Scellier and Bengio, 2017), we note that our rule is analytically equivalent to a modified version of DH that accounts for the specific architectural constraints as noted in the original work (see Appendix, Section 4.2).

As we conclude that there are multiple feedback-based learning models to which a DH learning rule generalizes, it is natural to inquire whether the specific combination of DH-DFC has any advantage over its predecessor. In the original studies of DFC, Predictive Coding, and Equilibrium Propagation, the learning rules are applied after the neural dynamics have converged to equilibrium. Learning is then based on an error-like component that corresponds to the difference between the activities (or membrane potentials) before and after feedback has shaped them. In DFC, this error is obtained by having two-compartment neurons, while in Predictive Coding errors are accumulated (usually as error neurons); in both cases, this raises the number of variables from *N* feedforward neurons to 2*N*. In contrast, both DH-DFC and Equilibrium Propagation rely on the same *N* neurons for the feedforward pass and the learning updates. However, Equilibrium Propagation requires a symmetry of the weights and weight updates, imposing a specific feedback architecture and an *ad-hoc* learning rule. In summary, we note that the DH-DFC is more parsimonious in the sense that it makes very simple assumptions on the feedback and requires less complex model architectures.

## 3. Discussion

Building upon previous studies, our work represents another leap forward to understanding the different aspects of hierarchical learning in biological networks. In the following sections, we go through the relationship between our work and previous works on computational and experimental neuroscience as well as limitations and future directions.

### 3.1. How does our work fit into the existing literature

A key contribution of our work is the connection between experimentally observed learning rules and computational models that can train deep networks. In this section, we discuss how this work fits with (1) the electrophysiology literature on learning rules, (2) temporal Hebbian learning rules both at the neuron and network level, (3) Predictive Coding and the combination of bottom-up inputs and top-down feedback, and (4) other bioplausible deep learning models.

#### 3.1.1. Electrophysiological observations that agree with our model

In biological neural networks, LTP and LTD are one of the most prevalent forms of synaptic plasticity, and various studies have shown that LTP is induced when presynaptic spikes precede postsynaptic ones. In the case of multiple spike pairs, this is consistent with our model in that an increase in postsynaptic activity would lead to LTP and a decrease in LTD. Interestingly, recent work suggests that classical STDP-inducing protocols might fail under physiological extracellular calcium concentrations, suggesting that additional mechanisms might be required to act on the intracellular calcium levels (Larkum et al., 1999; Inglebert et al., 2020). In pyramidal neurons, intracellular calcium levels can be modulated by backpropagating action potential-evoked calcium (BAC) spikes that arise when apical inputs arrive shortly after basal inputs, resulting in action potential bursts (Larkum et al., 1999). Our model is consistent with this notion that delayed feedback into the apical dendrite drives plasticity while basal feedforward input does not. Future neuroscience experiments should explore if high calcium concentrations resulting from BAC spikes and bursts are indeed suitable to restore LTP and LTD induction when using a classical STDP protocol (Inglebert et al., 2020).

Finally, our model requires feedback that is specific to every neuron. Therefore, the synaptic weights to the apical dendrite have very specific values that must be computed by some biological mechanism. In previous work, we showed that these weights can be learned in a bioplausible manner by an anti-Hebbian leaning rule (Meulemans et al., 2020). In biology, anti-Hebbian learning rules appear in disinhibitory GABAergic synapses (Lamsa et al., 2007), suggesting that the target used for learning in our model would be fed back into excitatory neurons through disinhibitory circuits. This nicely relates our work to the role of coupled apical and basal inputs in learning and the regulation of this coupling by disinhibitory circuits (Zhang et al., 2014; Avital et al., 2019; Williams and Holtmaat, 2019), and therefore use connectivity that matches the requirements of our feedback-based target propagation framework. Future theoretical investigations should continue this line of work by looking beyond Hebbian-like learning rules and integrating the knowledge of BAC-firing dynamics, the effects of calcium on plasticity, and the role of disinhibitory circuits in bioplausible models of deep learning.

#### 3.1.2. Learning with temporal Hebbian learning rules

Temporal Hebbian learning rules such as STDP or DH rules have been mostly used for unsupervised learning (Gerstner et al., 1996; Toyoizumi et al., 2005; Lazar et al., 2009; Sjöström and Gerstner, 2010) or as an enhancement of supervised learning in shallow networks (Diehl and Cook, 2015). In order to use these rules in a supervised setting, they require a teaching signal, which can be implemented either through a neuromodulator or a third-factor learning rule (Frémaux and Gerstner, 2016). However, such approaches do not go beyond shallow networks (Illing et al., 2019) and, although it has been suggested that STDP or DH could be adopted for error-driven hierarchical learning (Xie and Seung, 1999; Hinton, 2007; Bengio et al., 2017), a suitable network architecture and dynamics to combine time-dependent Hebbian learning rules with deep networks has not been proposed yet (Bengio et al., 2015). Our work fills this gap by presenting an approach that is able to train deep hierarchies with a learning rule that retains the time-based principles of STDP. This in turn connects deep network optimization to latency reduction, a well-known effect of STDP where neurons fire earlier in time every time that an input sequence is presented (Masquelier et al., 2009; Vilimelis Aceituno et al., 2020; Saponati and Vinck, 2021). This had been studied only at the level of neurons but we now turned it into a systems-level optimization process.

#### 3.1.3. Predictive Coding and top-down feedback

Due to the close relation of our model to Predictive Coding (PC), we next compare our approach to PC. In the PC literature, learning decreases the amount of top-down feedback. This process intrinsically generates expedited neuronal responses after stimulus presentation, which are often interpreted as predictions (Friston and Kiebel, 2009; Whittington and Bogacz, 2017; Keller and Mrsic-Flogel, 2018). The PC framework goes beyond explanations of these activities by proposing neural circuits that could implement this behavior (Rao and Ballard, 1999; Bastos et al., 2012).

**Figure 5:**
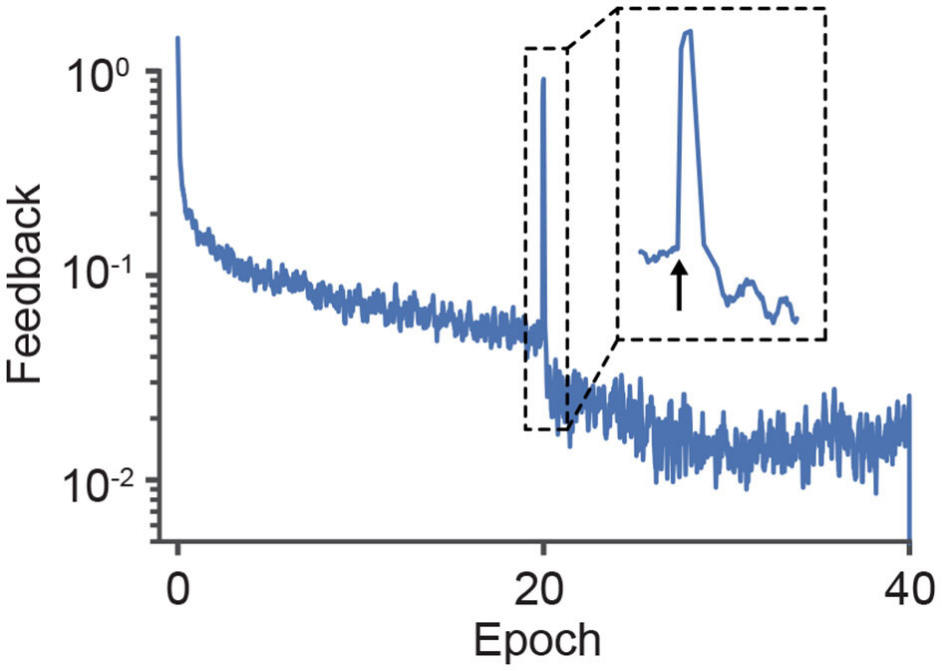
Surprise triggers a large feedback signal that alters neuronal activities. Across learning, the change in the post-synaptic activity driven by the apical feedback reduces as neurons reach their target rates. However, when the labels are randomly swapped (indicated by the arrow in epoch 20), the apical feedback is notably increased. Note that the label switch did not set the network to its baseline state, because the required feedback decreased to the pre-shift level much faster than on the first epochs.

However, PC as a mechanistic theory for neural circuits requires explicit error encoding (Koch and Poggio, 1999; Rao and Ballard, 1999; Bastos et al., 2012), a requirement which is problematic for making valid testable predictions (Kogo and Trengove, 2015). In contrast, our framework can exhibit a similar reduction of top-down feedback and anticipated neuronal responses. Still, since it is based on the target activities of neurons rather than on errors, it does not require explicit errors to be encoded. This shows that it is actually possible to design neural circuits that can reproduce the relevant PC features while representing errors implicitly with the temporal neuronal dynamics. To illustrate this effect, in Figure 5 we plot the feedback that modulates a deep network during training. The feedback decreases as the model learns but, when we randomly shuffle the labels—which can be considered a surprising response—the feedback signal increases substantially, thereby changing the neuronal activity in accordance with experimental observations (Keller and Mrsic-Flogel, 2018).

#### 3.1.4. Alternative bioplausible deep learning models

Other bioplausible deep networks models such as Equilibrium Propagation, dendritic-error learning, or Burst Propagation require a learning signal to be computed either by using two separate phases (Scellier and Bengio, 2017), distinct dendritic and somatic compartments (Sacramento et al., 2018) or via multiplexing of feedback and feedforward signals as bursts and single spikes (Payeur et al., 2021), respectively. In contrast, our model encodes supervision signals as temporal changes in postsynaptic activities, which arrive at individual neurons via their apical dendrite with a short time delay. Table 2 provides a comprehensive comparison of our approach to the most recent alternative bioplausible deep learning methods and how they relate to experimental observations.

**Table 2:** Comparison of diverse bioplausible hierarchical learning methods. For further details on these methods: Equilibrium Propagation (Scellier and Bengio, 2017), Predictive Coding (Whittington and Bogacz, 2017), Dendritic-error (Sacramento et al., 2018), Burst Propagation (Payeur et al., 2021).

	**Implicit error**		**Explicit error**		
**Method**	**Temporal Hebbian**	**Equilibrium Propagation**	**Predictive Coding**	**Dendritic-error**	**Burst Propagation**
Error type	Control	Contrastive	Prediction	Prediction	Multiplexed
Phases/Compartments	1/1	2/1	1/2	1/2	1/2
Network connectivity	Unconstrained	Unconstrained	Constrained	Constrained	Constrained
Weight symmetry	Not required	Required	Arises with learning	Not required	Arises with learning
Feedback magnitude	Strong feedback	Weak nudging	Weak error	Weak nudging	Weak error
Error encoding	Change in firing rate	Difference in neuronal activity	Activity of error neurons	Difference apical/ somatic activity	Ratio bursts/ single spikes
Update rule	Temporal Hebbian	Contrastive Hebbian	Hebbian	Apical/somatic error	Burst rate
Update timing	Continuous	Timed	Timed	Timed	Continuous
MNIST performance (%)	~1.9	~2–3	~1.7–1.8	~2.0	~1.1
Possible link to observations in neuroscience	Apical inputs affect postsynaptic activity, increased feedback activity upon new stimuli	Varying neural responses for different behaviors	Increased neural activity upon new stimuli	Apical inputs affect activity and plasticity	Apical inputs affect burst rate and plasticity
Bioplausibility	**+++**	**+**	**++**	**++**	**+++**

Note that the MNIST performance comparison does not take into account different model sizes. The +++ symbol corresponds to high biological plausibility, the ++ symbol corresponds to medium biological plausibility, and symbol corresponds the + to low biological plausibility.

The relationship between temporal dynamics and bioplausible deep learning has been explored before. This was done through different methods, for instance: by making use of subsequent frames, usually in an unsupervised or self-supervised setting (Illing et al., 2020; Lotter et al., 2020); or having a combination of STDP and reward signals (Mozafari et al., 2019; Illing et al., 2020); or, more generally, with the so-called temporal error learning framework (Wittenberg and Wang, 2006). Our model applies a similar principle but with a supervised target and at the level of neuronal dynamics.

### 3.2. Limitations and future work

#### 3.2.1. Limitations

On the experimental side, our framework requires a top-down controller to continuously compare the actual network output to the desired one, while sending feedback to the lower hierarchies. Although such a feedback controller can be easily realized as a neural circuit (Meulemans et al., 2021a), it is not clear yet if the brain employs any type of control circuit for learning. Future work could look at whether the apical inputs going through disinhibitory circuits correspond to feedback inputs that drive neurons to a target activity that stabilizes the top-down feedback.

From a modeling perspective, weights from the same neuron can be positive and negative or even transition from negative to positive and vice-versa, which is in conflict with Dale's law. This is a common simplification of ANN models (Cornford et al., 2020). Violating Dale's law, however, can be corrected using a bias in the postsynaptic activity to turn negative weights into weak positive weights (Kriegeskorte and Golan, 2019). Moreover, recent studies showed that with certain network architectures and priors, Dale's law can be easily preserved while maintaining the same functional network properties (Cornford et al., 2020).

Another limitation of our work is that we use DH instead of STDP to train deep networks. This is due to the randomness induced by our implementation of spiking neurons using a Poisson model, which implicitly imposes noisy learning updates. Further work could use leaky integrate-and-fire neurons, which can reduce the effects of randomness. This would require computing feedback in an event-based network, which is a currently active area of research.

At the computational level, our method requires a long time to be simulated because the controller works by updating the neural activity in small incremental steps, requiring as many as a hundred forward passes for each sample, which is much more than off-the-shelf learning algorithms but in line with previous works using feedback mechanisms (Scellier and Bengio, 2017; Rosenbaum, 2022). Similarly to the previous point, the use of an event-based network would greatly reduce the computational costs of learning by reducing the control cost only to relevant events.

#### 3.2.2. Future work

After learning, our model predicts an expedited onset of pyramidal neuron activity upon feedforward input (Figure 3) that is inversely correlated with the top-down feedback to alter neuronal activity. A related cortical micro-circuit hypothesis is that local inhibitory microcircuits projecting onto apical dendrites control the neuron's excitability and that their control strength reduces during learning. In an experimental setting, this temporal shift as well as the feedback strength attenuation could be tested using simultaneous *in vivo* 2-photon calcium imaging of excitatory and inhibitory populations (as in Han et al., 2019) combined with a plasticity-inducing whisker stimulation paradigm.

From a computational perspective, follow-up studies should go beyond modeling phenomenological learning rules such as STDP into hierarchical networks. For example, one direction could be to develop a more detailed mechanistic sub-cellular model that accounts for the coupling of intracellular voltage and calcium dynamics that are being differently modulated by inputs to apical and somatic synapses. Such sub-cellular mechanistic models might also include multiplicative effects of the apical input (Larkum et al., 2004) as well as apical-induced bursting (Segal, 2018) to further close the gap between the correlation-based models used in computational neuroscience and experimental observations showing, for example, the diverse intracellular effects of calcium on learning and neuronal activity (Larkum et al., 1999, 2007; Larkum, 2013). Another logical future step would be to develop more explicit theoretical links between PC and our temporal Hebbian framework. This would require applying it to other problems, such as detecting deviations from learned time series (Garrido et al., 2009) or unsupervised image representations (Rao and Ballard, 1999) and comparing the reduction of feedback with the minimization of prediction errors or free energy (Friston and Kiebel, 2009). Showing such conceptual links would pave the way to design more cortical-like circuits that explain Predictive Coding features but avoid the problems emerging from explicit error neurons (Kogo and Trengove, 2015).

Our framework can be leveraged to build the theory in spiking neural networks, where the processing of time-centered losses is still in its infancy. For example, it would be interesting to see how the notion of control cost or latency to target response interplay with information theory metrics, which have been shown to be useful for continuous learning or few-shot learning (Yang et al., 2022b,c). Similarly, if using the multi-compartment neuron formulation of our model, one could include other relevant features such as working memory (Yang et al., 2022a).

Finally, the simplicity and locality of the model we propose makes it well-suited for on-chip event-based learning applications. This would require integrating a simple PI controller in a neuromorphic processor and further theoretical work on implementing our learning set-up with leaky integrate-and-fire neurons. Given that STDP can induce energy-efficient representations (Vilimelis Aceituno et al., 2020), it is likely that training with STDP might even further improve the energy efficiency of neuromorphic devices. In addition, the fact that our framework can learn all weights in an online manner (Meulemans et al., 2021b) implies that a perfect model of the processor architecture is not required, which is a key problem when training neuromorphic devices off-line due to the so-called device mismatch (Pelgrom et al., 1989; Binas et al., 2016).

## 4. Conclusions

With this work, we present a new hierarchical learning framework in which the temporal order of neuronal signals is leveraged to encode top-down error signals. This reformulation of the error allows us to avoid unobserved learning rules while at the same time being consistent with classical ideas of Predictive Coding. Our work is a crucial step toward a more detailed understanding of how temporal Hebbian and STDP learning can be used for supervised learning in multilayer neural networks.

## Data availability statement

The code repository is publicly available in GitHub: https://github.com/MatildeTristany/Learning-Cortical-Hierarchies-with-Temporal-Hebbian-Updates.

## Author contributions

PA, MF, and BG designed the project and the experiments and wrote the paper. PA developed the mathematical STDP framework and performed the single neuron and Predictive Coding simulations. MF performed the network simulations. RL provided biology insights about the project on both theoretical and experimental neuroscience and wrote part of the discussion section.
