# Supplementary material for: Learning cortical hierarchies with temporal Hebbian updates
Source: Front Comput Neurosci. 2023 May 24;17:1136010. doi: 10.3389/fncom.2023.1136010 (PMC10244748; doi:10.3389/fncom.2023.1136010)
Supplement: Supplementary file 1 [file Data_Sheet_1.pdf]

APPENDIX

I Background

In this section, we explain the background concepts that are necessary to understand our work.

I.1 Poisson neurons

The neurons used in Section 2.1 are Poisson neurons, which correspond to inhomogeneous Poisson point processes in the temporal line where the rate is the firing rate of the neuron. In practice, those are implemented by first calculating the firing rate $r_{\text{post}}(t)$ as described in Eq. 1, and then, at every time-step in our simulations, we generate a spike with probability $r_{\text{post}}(t)$.

I.2 Spike-timing-dependent plasticity

In the single neuron example, we use the STDP learning rule across all pairs of spikes, which can be written for a single synapse (Sjöström et al., 2010) as

$$\Delta w_{nm} = \sum_{s_{\text{pre}}} \sum_{s_{\text{post}}} f(t_{s_{\text{post}}} - t_{s_{\text{pre}}}) \tag{9}$$

where $s_{\text{post}}, s_{\text{pre}}$ are the pre- and post-synaptic spikes, $t_{s_{\text{post}}}, t_{s_{\text{pre}}}$ their respective times, f is the function

$$\begin{aligned} f(\Delta t) &= -\eta e^{\frac{\Delta t}{\tau_{STDP}}} \quad \iff \Delta t < 0 \\ f(\Delta t) &= \eta e^{\frac{\Delta t}{\tau_{STDP}}} \quad \iff \Delta t > 0 \end{aligned} \tag{10}$$

where η is the learning rate and τ_{STDP} is the time constant of the STDP learning rule.

II Cost functions for Differential Hebbian in single layer networks

In this section, we show that the DH rule minimizes the cost functions mentioned in the main text. Even though we are referring to the simple neuron example represented in Figure 2, the results naturally hold for any single-layer linear classification setting.

II.1 MSE loss as a cost function

For the MSE loss cost function, $\mathcal{L}$, we note that the presynaptic activity is fixed, giving us the update rule

$$\Delta^e w \propto \int \dot{r}^e_{\text{post}}(t) \times r^e_{\text{pre}} dt = \Delta^e r_{\text{post}} \times r^e_{\text{pre}} = \left(r^{T,e}_{\text{post}} - r^{FF,e}_{\text{post}} \right) \times r^e_{\text{pre}}, \tag{11}$$

where $w = (w_A, w_B)$, $\Delta w = (\Delta w_A, \Delta w_B)$, r^e_{pre} is the presynaptic activity vector, and e corresponds to the training example. For that specific example, the loss can then be represented as

$$\mathcal{L}^e = \left(r^{T,e}_{\text{post}} - r^{FF,e}_{\text{post}} \right)^2, \tag{12}$$

and using a standard derivative on the weights w_A, w_B we recover the update rule in Eq. 11,

$$\frac{\partial \mathcal{L}^e}{\partial w} = -2\left(r_{\text{post}}^{T,e} - r_{\text{post}}^{FF,e}\right)\frac{\partial r_{\text{post}}^{FF,e}}{\partial w} = -2\left(r_{\text{post}}^{T,e} - r_{\text{post}}^{FF,e}\right) \times r_{\text{pre}}, \tag{13}$$

which corresponds to a standard gradient descent update.

To show that the feedback, $\mathcal{H}$, and time to target, $\mathcal{T}$, are also minimized, we define $r_{\text{post}}^{e}(t)$ as a dynamical system whose attractive fixed point is the target activations for the different labels. Each example provides one initial condition of the dynamical system and the feedback ensures that the dynamics converge to the desired target.

II.2 Feedback strength as a cost function

In order to make the transition to the feedback strength, $\mathcal{H}$, we note that

$$\phi^{-1}\left(r_{\text{post}}^{e,t}\right) = c^{T,e} + w_A r_A + w_B r_B, \tag{14}$$

where $\phi^{-1}\left(r_{\text{post}}^{e,t}\right)$ is the required membrane potential for the postsynaptic neuron to reach its target, hence, it is fixed while the right-hand side changes by learning. This allows us to rewrite the feedback strength cost function as

$$\mathcal{H} = \left(c^{T,e}\right)^2 = \left(\phi^{-1}\left(r_{\text{post}}^{e,T}\right) - v_{\text{post}}^{e}(0)\right)^2 = \left(\phi^{-1}\left(r_{\text{post}}^{e,T}\right) - (w_A r_A + w_B r_B)\right)^2, \tag{15}$$

and, thus, we can write the gradient of the feedback strength cost function as

$$\frac{\partial \mathcal{H}^e}{\partial w} = -2\left(\phi^{-1}\left(r_{\text{post}}^{e,T}\right) - (w_A r_A + w_B r_B)\right) \times r_{\text{pre}} = \frac{\left(r_{\text{post}}^{T,e} - r_{\text{post}}^{FF,e}\right) \times r_{\text{pre}}}{\phi'(v_m^e)} = \frac{1}{\phi'(v_m^e)}\frac{\partial \mathcal{L}^e}{\partial w}, \tag{16}$$

where the factor $\phi'(v_m^e) \geq 0$ with $v_m^e \in \left[w_A r_A + w_B r_B, \phi^{-1}\left(r_{\text{post}}^{e,T}\right)\right]$ comes from applying the mean value theorem to the firing rate as a (positive definite) function of the membrane potential. Note that this is a very similar statement to the one presented in Meulemans et al. (2021a).

II.3 Time-to-target as a cost function

In order to prove that the time delay cost function $\mathcal{T}$ is minimized, we need to make assumptions on the feedback, since the trajectory and speed depend on which controller is used. Specifically, we will assume that the calculation of the feedback works directly on $v_{\text{post}}^{e}(t)$, forcing it to reach its target value $v_{\text{post}}^{e,T}$. We will also assume that the feedback is computed by a PI controller, which stably converges to its target.

Since feedback pushes the firing rate to a target value, the dynamics of the membrane potential $v_{\text{post}}^{e}(t)$ converge to a stable fixed point $v_{\text{post}}^{e,T} = \lim_{T\to\infty} v_{\text{post}}^{e}(T)$. In order to avoid the limit $T \to \infty$, we will consider the state space as a ball of radius ϵ centered around the target, and that the network has converged when it reaches that ball.

In a linear stable dynamical system, the activity evolves according to

$$v_{\text{post}}^{e}(t) = v_{\text{post}}^{e}(T) + \left[v_{\text{post}}^{e,T} - v_{\text{post}}^{e}(0)\right] e^{-pt}, \tag{17}$$

where p is the rate of convergence given by the projection of $\left[v_{\text{post}}^{e,T} - v_{\text{post}}^{e}(0)\right]$ on the eigenspace of the feedback, which is positive as long as the feedback is stable.

Given the activity evolution, we can compute the time it would take for a training example to reach the target, τ^{ϵ}, by

$$\tau^{\epsilon} = \frac{1}{p} \log \left(\frac{\|v_{\text{post}}^{e,T} - v_{\text{post}}^{e}(0)\|}{\epsilon} \right). \tag{18}$$

We note that if the system is nonlinear but monotonic, as it would be if the feedback is computed using the firing rate $r_{\text{post}}^{e}(t) = \phi\left(v_{\text{post}}^{e}(t)\right)$, the previous formula would not work. Then, we would have to consider the projection $p_{r_{\text{post}}^{e}(t)}$ as a state-dependent exponent. While this would not have a closed-form solution, it can be bounded through the Lyapunov exponents of the network, in which case we recover the current formula with a scalar that depends on the nonlinearity.

III Extension to multilayer networks

In this section, we argue that the DH learning rule allows learning in multilayer networks for all the loss functions discussed in the previous section.

The gist of our argument is that the feedback defines a target activity for all layers of the network. Each layer has its own cost function, and the global cost function of the network is a positive semi-definite composition of the layerwise cost functions. Hence, global optimization is guaranteed by learning in each layer. Note that, although the DFC framework provides a specific target, many different possible targets could be used with this model (Meulemans et al., 2020); Here, we will consider only one target for simplicity and ignore this multiplicity.

III.1 Differential Hebbian Learning learns the target

In this section, we show that given a feedback signal that successfully pushes the whole network to its target activities $r^{e,T} = \left[r_0^T, ...r_l^T, ..., r_L^T\right]$, the Differential Hebbian learning rule has $r^{e,T}$ as the attractive fixed point of the learning process. In mathematical terms

$$\Delta^e w_l = 0 \iff r_l^{T,e} = r_l^{FF,e}. \tag{19}$$

We start with the first layer. Given that the input is static and has a predefined target activity $r_1^{e,T}$, then

$$\Delta^e w_1 \propto \int \dot{r}_1^e(t) \times r_0^e dt = \Delta r_1^e \times r_0^e = \left(r_1^{T,e} - r_1^{FF,e}\right) \times r_0^e, \tag{20}$$

which has a single attractive fixed point for the learning dynamics at $r_1^e(0) = r_1^{T,e}$.

Now, we can proceed by induction. For any layer $l+1$, the DH learning rule will give the following weight update

$$\Delta^e w_{l+1} \propto \int \dot{r}^e_{l+1}(t) \times r^e_l dt = \int \dot{r}^e_{l+1}(t) \times r^{e,T}_l dt - \int \dot{r}^e_{l+1}(t) \times \tilde{r}^e_l(t) dt$$
$$= \left(r^{T,e}_{l+1} - r^{FF,e}_{l+1} \right) \times r^{e,T}_l - \int \dot{r}^e_{l+1}(t) \times \tilde{r}^e_l(t) dt, \tag{21}$$

where $\tilde{r}^e_l(t) = r^{e,T}_l - r^e_l(t)$ is the difference between the firing rate in layer l at time t and its target.

After enough learning, we have that $r^e_l(0) = r^{e,T}_l$. This implies that there is some epoch after which the learning rule in Eq. 21 becomes

$$\Delta^e w_{l+1} \propto \left(r^{T,e}_{l+1} - r^{FF,e}_{l+1} \right) \times r^{e,T}_l. \tag{22}$$

Which will eventually converge, giving us $r^e_{l+1}(0) = r^{e,T}_{l+1}$. This can be iterated for all layers.

Note that the calculations presented are based on a single example, so the fixed point relates to a single class or label. Having an underparametrized model implies that there will be trade-offs such as the fixed point of the network parameters will not reaching zero loss for every example. How the loss for different examples is combined into a global loss in underparametrized systems is unknown in standard BP, Predictive Coding, Equilibrium Propagation, or DFC, as it is in our setup.

III.2 Global and layerwise loss functions in deep networks

In this section, we define a loss function for every layer of the neural network and show that the global cost function is a positive definite composition of those rules.

We consider the cost functions for a given layer l denoted as $\mathcal{L}_l, \mathcal{H}_l, \mathcal{T}_l$. All of those will be defined on one layer assuming that the input to that layer is fixed to its target activity. We will also assume that the feedback is given by an oracle that knows from the beginning what the target activity of each neuron is and exactly the required feedback for the neuron to remain fixed to its target when the rest of the network is at equilibrium. Although this might be practically unfeasible in nonlinear systems, in practice, the appropriate PI controllers can provide good approximations.

First, we start with the $\mathcal{L}$ global cost function. It is clear that the error is given by the last layer, so $\mathcal{L} = \mathcal{L}_L$. Second, we consider the $\mathcal{H}$ global cost function. The total amount of feedback that is given to the network is the sum of the amount of feedback given to each layer, so $\mathcal{H} = \sum_l \mathcal{H}_L$. Finally, for the time-to-target, $\mathcal{T}$, the final layer can reach an equilibrium only if all the layers have reached theirs. Hence, this takes as long as the slowest layer, so $\mathcal{T} = \max_l \left[\mathcal{T}^e_l, ...\mathcal{T}^e_l, \mathcal{T}^e_L \right]$.

From Section II, we know that reaching the fixed point of DH learning minimizes the local cost functions $\mathcal{L}_l, \mathcal{H}_l, \mathcal{T}_l$ if the presynaptic activity is static. By applying the inductive argument from Section 2.2, we know that throughout the learning the presynaptic activities will be approximately static, so the layerwise loss functions will be minimized. Finally, since the global losses are a positive definite combination of the layerwise losses, minimizing the local losses also leads to a minimization of the local ones.

IV Differential Hebbian learning works with other feedback-based deep learning frameworks

Our work relies on the DFC framework as the underlying model, but it is worth noting that the principles outlined here apply to other models. To make this point, we will show analytically that the DH learning rule we used approximates the learning updates of the DFC as well as other bioplausible learning models that have been used to train deep neural networks. We restrict ourselves to supervised models for comparability.

IV.1 Dendritic error rule from DFC

The dendritic error learning rule has been used in previous works including the DFC with both strong and weak feedback (Meulemans et al., 2021a, 2022a), as well as in previous implementations of BP using specific circuits (Sacramento et al., 2018), although for simplicity we focus on the DFC framework. In this framework, feedback affects neuronal activity until the later converges to a steady state (thus ignoring time), and each neuron has therefore two membrane potentials for its two compartments:

$$\begin{aligned} \mathbf{v}^{\text{ff}} &= W\mathbf{r} \\ \mathbf{v} &= \mathbf{v}^{\text{ff}} + Q\mathbf{c} \end{aligned} \tag{23}$$

Then, the weight update uses the two-compartment neuron model, the weight update for layer l being given by

$$\Delta_{DFC} W_{l+1} \propto \left(\mathbf{v}_{l+1} - \mathbf{v}_{l+1}^{\text{ff}}\right) \times \mathbf{r}_l. \tag{24}$$

The first difference compared to the DH learning rule is that DH uses the postsynaptic firing rates instead of the membrane potentials. However, given that the nonlinearity is monotonic this consists of a simple scaling, which we can then ignore.

Another more subtle difference is that the postsynaptic term in Eq. 24 at the neuron level consists of the difference between the state of the neuron with feedback $\mathbf{v}$ and the state of the neuron without feedback *but with all presynaptic neurons having feedback* $\mathbf{v}^{\text{ff}}$. In contrast, for the DH learning rule from Eq. 22, the term without feedback $\mathbf{r}_{l+1}^{FF}$ consists of the activity of the neuron *before any feedback is given in the whole network*, and it also has the extra term $-\int \dot{r}_{l+1}(t) \times \tilde{r}_l(t)dt$.

Following the logic of Meulemans et al. (2021a), we compare the two rules by looking at their fixed points in weight space. Thus, in the proximity of the fixed point where $\Delta_{DFC} W_l \approx 0$, the activity of the presynaptic layer has a very small change

$$\phi(\mathbf{v}^{\text{ff}}) \approx \mathbf{r}_{l+1}^{FF}, \tag{25}$$

and for the same reason $\tilde{r}_l(t) \approx 0$ and, thus, the second term disappears. Then, we can conclude that the two rules have the same fixed points.

IV.2 Predictive Coding learning rules

Previous work (Whittington and Bogacz, 2017) has shown that Predictive Coding (Rao and Ballard, 1999) can be adapted to implement an approximation of the BP algorithm. Here, we show that, under assumptions made in the original work, an approximation of the weight updates given by their algorithm can be formulated as DH learning.

A full description of that work is beyond the scope of this paper, but we will briefly outline the logic. First, the input is presented to the network, and the activity of the neurons is propagated through each layer in the same manner as ours:

$$\mathbf{r}_{l+1}(0) = \phi\left[W_l \mathbf{r}_l(0)\right], \tag{26}$$

where the neurons in layer l are the presynaptic neurons to layer $l+1$ and the zeroth layer corresponds to the input. Second, an error at the output layer is computed as the difference between the current output and the target output. Using a quadratic error as originally done,

$$\varepsilon_L = \frac{\partial \mathcal{L}}{\partial \mathbf{r}_L(0)} = \mathbf{r}_L^T - \mathbf{r}_L(0). \tag{27}$$

Third, this error is used to update the hidden layers by

$$\begin{aligned} \varepsilon_l(t) &= \mathbf{r}_l(0) - \mathbf{r}_l(t) \\ \dot{\mathbf{r}}_l(t) &= \varepsilon_l(t) - \varepsilon_{l+1} \frac{\partial \phi\left(W_l \mathbf{r}_l(0)\right)}{\partial \mathbf{r}_l(0)}. \end{aligned} \tag{28}$$

By construction, the update of the hidden layers pushes their initial activity, $\mathbf{r}_l(0)$, to their target activity, $\mathbf{r}_l^T = \mathbf{r}_l(T)$, which reduces ε_{l+1}. Finally, the weights are updated with

$$\Delta_{PC} W_{l+1} = -\varepsilon_{l+1} \frac{\partial \phi\left(W_{l+1} \mathbf{r}_l\right)}{\partial W_{l+1}}. \tag{29}$$

The definition of errors in Eq. 28 after convergence $\varepsilon_l = \mathbf{r}_l(0) - \mathbf{r}_l(T)$ can be inserted into the weight update from Eq. 29, giving us

$$\Delta_{PC} W_{l+1} = \left(\mathbf{r}_{l+1}(T) - \mathbf{r}_{l+1}(0)\right) \frac{\partial \phi\left(W_{l+1} \mathbf{r}_l(0)\right)}{\partial W_{l+1}}. \tag{30}$$

By using the chain rule to expand the partial derivative above, we find that

$$\Delta_{PC} W_{l+1} = \left[\left(\mathbf{r}_{l+1}(T) - \mathbf{r}_{l+1}(0)\right) \cdot \phi'\left(\mathbf{v}_{l+1}\right)\right] \times \mathbf{r}_l(0), \tag{31}$$

where $\cdot$ is the element-wise product. Furthermore, as in the original work (Whittington and Bogacz, 2017) the activities are only nudged (meaning slightly changed), then we can assume $\mathbf{r}_l(T) - \mathbf{r}_l(0) \ll \mathbf{r}_l(T)$, and so

$$\Delta_{PC} W_{l+1} \approx \left[\left(\mathbf{r}_{l+1}(T) - \mathbf{r}_{l+1}(0)\right) \cdot \phi'\left(\mathbf{v}_{l+1}\right)\right] \times \mathbf{r}_l(T). \tag{32}$$

At this point, it is worth writing how the weight update would have looked if we used the DH learning rule

$$\Delta_{PC-DH} W_{l+1} = \int \dot{\mathbf{r}}_{l+1}(t) \times \mathbf{r}_l(t) dt = \left(\mathbf{r}_{l+1}(T) - \mathbf{r}_{l+1}(0)\right) \times \mathbf{r}_l(T) \tag{33}$$

$$+ \int \dot{\mathbf{r}}_{l+1}(t) \times \tilde{\mathbf{r}}_l(t) dt \approx \left(\mathbf{r}_{l+1}(T) - \mathbf{r}_{l+1}(0)\right) \times \mathbf{r}_l(T), \tag{34}$$

where the approximation is due to the weak nudging assumption which states $\tilde{\mathbf{r}}_l(t) \ll \mathbf{r}_l(T)$. Looking at the original rule of Predictive Coding and its DH formulation, the only difference is the term $\phi'\left(\mathbf{v}_{l+1}\right)$,

which is positive for all monotonically increasing nonlinearities (such as $\tanh$ or ReLu), implying that the synaptic updates are positively correlated, that is, that the weight updates are aligned.

Therefore, the DH learning rule that we used in our model should be compatible with the error-based learning rule of the Predictive Coding implementation of Whittington and Bogacz (2017).

IV.3 Equilibrium Propagation

Another influential model in bioplausible deep learning is Equilibrium Propagation (Scellier and Bengio, 2017), where the network has symmetric weights and the activities at the output and the neuronal dynamics alternate between free and weakly nudged output neurons. The connection with differential Hebbian learning was already noted in the original work, but to make the point clear we go over it here as well.

As before, we briefly outline the logic of Equilibrium Propagation, but for simplicity, we avoid the energy formalism and concentrate on the neuronal dynamics, which can be decomposed into two phases, one where the output is free, that is not clamped (equivalent to a forward pass in deep networks), and one where the output is weakly nudged towards the right value (equivalent to a feedback pass).

The neuronal dynamics of the network are given by

$$\dot{\mathbf{v}}_{\text{post}}(t) = -\mathbf{v}_{\text{post}}(t) + W_{\{\text{pre,post}\}}\mathbf{r}_{\text{pre}}(t) + \beta\left(\hat{y} - y\right), \tag{35}$$

where y is the state of the output neuron, $\hat{y}$ is its target state, and β (originally set to be a small positive value) is the strength with which the output neurons are nudged towards the targets.

The learning rule then compares the firing rates of the pre- and post-synaptic neurons before and after the nudging. In our notation, the firing rate of the neurons before nudging is $\mathbf{r}(0)$, and after the nudging, it corresponds to the dynamics already at equilibrium, so $\mathbf{r}(T)$. More explicitly,

$$W_{\{\text{pre,post}\}} \propto \left[\mathbf{r}_{\text{pre}}(T)\mathbf{r}_{\text{post}}(T) - \mathbf{r}_{\text{pre}}(0)\mathbf{r}_{\text{post}}(0)\right]. \tag{36}$$

By using integration by parts in reverse, we can simplify this as

$$\left[\mathbf{r}_{\text{pre}}(T)\mathbf{r}_{\text{post}}(T) - \mathbf{r}_{\text{pre}}(0)\mathbf{r}_{\text{post}}(0)\right] = \int_0^T \mathbf{r}_{\text{pre}}(t)\mathbf{r}_{\text{post}}(t)dt \tag{37}$$

$$= \int_0^T \dot{\mathbf{r}}_{\text{pre}}(t)\mathbf{r}_{\text{post}}(t)dt + \int_0^T \mathbf{r}_{\text{pre}}(t)\dot{\mathbf{r}}_{\text{post}}(t)dt. \tag{38}$$

Since Equilibrium Propagation requires the assumption of weight symmetry (feedforward weights are the same as feedback weights), then its weight updates must also be symmetric and, hence, Eq. 37 represents the symmetric version of the DH learning rule (Scellier and Bengio, 2017).

V STDP as a noisy version of Differential Hebbian

Although DH is supposedly equivalent to STDP with the right weight update kernel (Zappacosta et al., 2018), we found in Figure 4 that the weight updates with STDP are very noisy. We argue that the reason for this is that the conversion from rates – which we use to compute the appropriate feedback – to spike trains – which are required for STDP – induces randomness due to the use of a Poisson neuron model. A Poisson neuron model is by nature random, and we would expect some level of noise to be present all throughout the training. However, while the noise might remain at a given level, the learning signal does not; as the

network is trained, the neurons start very close to their final targets, and therefore the change in neuronal activity – the learning signal – becomes much smaller. Maintaining a fixed level of noise but decreasing the signal strength implies that the learning signal eventually gets drowned in noise and cannot reach the level of precision necessary for competitive deep learning performances.

To test this argument we note that if every conversion from firing rates to spikes is noisy, we can reduce the noise by averaging for many conversions. Thus, we run our deep network for each epoch, but for every input, we perform multiple conversions of spike trains to firing rates and then average over the resulting STDP weight updates. We see that averaging indeed increases the performance achieved by the network (see Fig. S1). However, there is a limit to the feasibility of the approach, in that the number of averages required grows exponentially, thus scaling it up to reach state-of-the-art machine learning performance is not feasible.

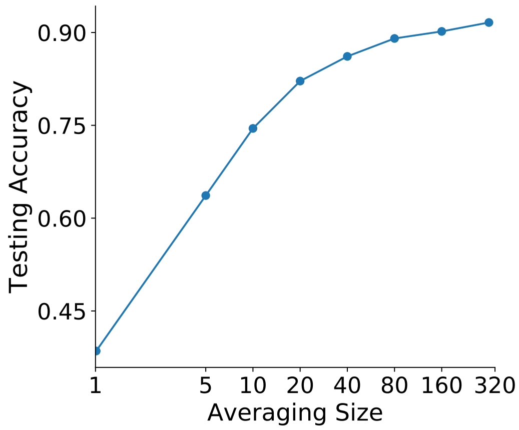

Figure S1. Averaging STDP increases its performance. We train a network with size 256x256x256 for the MNIST task but instead of using the DH learning rule we convert the rates to spikes with a Poisson neuron model and use the STDP learning rule. We observe a steady increase in performance with the number of conversion samples.

VI Simulations

VI.1 Description of the hyperparameter searches

The set of hyperparameters chosen for reporting values was selected by the best validation accuracy of all the training epochs, using 5000 validation samples extracted from the training set. We use the Tree of Parzen Estimators hyperparameter optimization algorithm (Bergstra et al., 2011) based on the Hyperopt (Bergstra et al., 2013) and Ray Tune (Liaw et al., 2018) Python libraries.

VI.2 Description of training

Weights and neurons initializations: The feedforward network weights are initialized with the Glorot-Bengio normal initialization (Glorot and Bengio, 2010). Unlike in (Meulemans et al., 2021a,b), the feedback weights here are kept frozen throughout the training and initialized to pre-trained values.

Activation functions: For the hidden neurons, we use the sigmoid activation and for the output neurons, we use a linear activation with a softmax readout.

Optimizer: To perform the reported MNIST experiments, we use Adam optimizer (Kingma and Ba, 2014) for the forward weights, which improves results compared to vanilla SGD. As mentioned in Meulemans et al. (2021a), Adam was designed for BP updates, so it is most likely not an optimal optimizer for DFC, which uses MN updates.

Differential Hebbian updates computation: We compute the DH updates throughout the time series of the pre- and post-synaptic firing rates. We compute the postsynaptic change in activity at each timestep and then multiply the result by the corresponding presynaptic activity,

$$\Delta_{\text{DH}} w = \sum_{t=0}^{T-1} r_{\text{pre}}(t) \left(r_{\text{post}}(t+1) - r_{\text{post}}(t) \right) . \tag{39}$$

STDP updates computation: We compute the STDP updates by considering the time series of the pre- and post-synaptic rates. First, we convert the rates into spike trains by sampling from a random uniform distribution in the interval $[0, 1]$ and comparing it with the firing rate at each timestep. Then, we convolve the presynaptic spike train with the following STDP kernel

$$\kappa(t) = e^{-\frac{t}{\tau_s}}\Theta\,[t] + e^{\frac{t}{\tau_s}}\Theta\,[-t]\,, \tag{40}$$

where $\Theta\,[t]$ is the step function and we set $\tau_s = 9.5$ so that the decay rate is $e^{-\frac{1}{\tau_s}} = 0.9$ (for Fig. 2). Finally, we compute the weight update as the dot product of the convolved presynaptic spike train and the postsynaptic spike train,

$$\Delta_{\text{STDP}} w = \sum_{t=0}^{T} \left[s_{\text{pre}} * \kappa\right](t), s_{\text{post}}, \tag{41}$$

where $s_{\text{pre/post}}(t)$ is a spike train encoded as a vector of zeros and ones and $*$ is the convolution operator.

VI.3 Description of the measures

Here, we describe how the measures reported in Figure 3 were obtained:

- **Feedback:** the feedback is measured as the amount of apical signal sent to each neuron, represented by $c(t)$ Eq. 1. We report the average feedback strength per neuron across layers throughout the training epochs as the L2 norm (as in Meulemans et al. (2021b)).
- **MSE loss:** the mean squared error loss is computed taking the network's output without feedback and the true labels throughout the training epochs.
- **Time-to-target:** the time-to-target was computed using DH learning with the original weak feedback setting of DFC (Meulemans et al., 2021a) as otherwise, the strong feedback setting (Meulemans et al., 2021b) immediately pushes the network's output to the correct targets. For this, we measure the number of time steps taken to reach an ϵ-distance to the correct targets, where ϵ was taken as the maximum distance between the network's output and the output targets for the last epoch.

VI.4 Resources and compute

To perform the reported MNIST experiments, we used GeForce RTX 2080 and GeForce RTX 3090 GPUs. We run for 40 training epochs and we did hyperparameter searches of 200 samples as in Meulemans et al. (2021a). The BP and DFC results are the same as reported in Meulemans et al. (2021b).

VI.5 Datasets and code licenses

For the experiments reported in this paper, we used the MNIST dataset (LeCun, 1998), with the license `https://creativecommons.org/licenses/by-sa/3.0/`.

For the implementation of the model, we used PyTorch (Paszke et al., 2019) and built upon the codebase of Meulemans et al. (2020, 2021a,b), which has the following licenses:

- Pytorch: `https://github.com/pytorch/pytorch/blob/master/LICENSE`
- Meulemans et al. (2020): `https://www.apache.org/licenses/LICENSE-2.0`

The PC and PC-DH were based on the code provided by Tschantz (2020), a Python implementation of the original model (Whittington and Bogacz, 2017). The license can be found in `https://opensource.org/license/mit/`.

REFERENCES

Abbott, L. F. and Nelson, S. B. (2000). Synaptic plasticity: taming the beast 3, 1178–1183. doi:10.1038/81453

Avital, A., Ruohe, Z., Myung, E. S., Ryohei, Y., and Gan, W.-B. (2019). Somatostatin-expressing interneurons enable and maintain learning-dependent sequential activation of pyramidal neurons. *Neuron* 102, 202–216

Bastos, A. M., Usrey, W. M., Adams, R. A., Mangun, G. R., Fries, P., and Friston, K. J. (2012). Canonical microcircuits for predictive coding. *Neuron* 76, 695–711

Bengio, Y., Lee, D.-H., Bornschein, J., Mesnard, T., and Lin, Z. (2015). Towards biologically plausible deep learning. *arXiv preprint arXiv:1502.04156*

Bengio, Y., Mesnard, T., Fischer, A., Zhang, S., and Wu, Y. (2017). Stdp-compatible approximation of back-propagation in an energy-based model. *Neural Computation*

Bergstra, J., Yamins, D., and Cox, D. D. (2013). Hyperopt: A python library for optimizing the hyperparameters of machine learning algorithms. In *Proceedings of the 12th Python in science conference* (Citeseer), 13–20

Bergstra, J. S., Bardenet, R., Bengio, Y., and Kégl, B. (2011). Algorithms for hyper-parameter optimization. In *Advances in neural information processing systems*. 2546–2554

Bi, G.-q. and Poo, M.-m. (1998). Synaptic modifications in cultured hippocampal neurons: Dependence on spike timing, synaptic strength, and postsynaptic cell type 18, 10464–10472. doi:10.1523/JNEUROSCI.18-24-10464.1998

Binas, J., Neil, D., Indiveri, G., Liu, S.-C., and Pfeiffer, M. (2016). Precise deep neural network computation on imprecise low-power analog hardware. *arXiv: Computer Science/Neural and Evolutionary Computing* 1606, 0–0

Cornford, J., Kalajdzievski, D., Leite, M., Lamarquette, A., Kullmann, D. M., and Richards, B. (2020). Learning to live with dale's principle: Anns with separate excitatory and inhibitory units. *bioRxiv* doi:10.1101/2020.11.02.364968

Crick, F. (1989). The recent excitement about neural networks. *Nature* 337, 129–132

Diehl, P. U. and Cook, M. (2015). Unsupervised learning of digit recognition using spike-timing-dependent plasticity. *Frontiers in computational neuroscience* 9, 99

Feldman, D. (2012). The spike-timing dependence of plasticity 75, 556–571. doi:10.1016/j.neuron.2012.08.001

Frémaux, N. and Gerstner, W. (2016). Neuromodulated spike-timing-dependent plasticity, and theory of three-factor learning rules. *Frontiers in neural circuits* 9, 85

Friston, K. and Kiebel, S. (2009). Predictive coding under the free-energy principle. *Philosophical transactions of the Royal Society B: Biological sciences* 364, 1211–1221

Garrido, M. I., Kilner, J. M., Stephan, K. E., and Friston, K. J. (2009). The mismatch negativity: a review of underlying mechanisms. *Clinical neurophysiology* 120, 453–463

Gerstner, W., Kempter, R., van Hemmen, J. L., and Wagner, H. (1996a). A neuronal learning rule for sub-millisecond temporal coding. *Nature* 383, 76

Gerstner, W., Kempter, R., van Hemmen, J. L., and Wagner, H. (1996b). A neuronal learning rule for sub-millisecond temporal coding 383, 76–78. doi:10.1038/383076a0

Gilbert, C. D. and Li, W. (2013). Top-down influences on visual processing. *Nature Reviews Neuroscience* 14, 350–363

Gilra, A. and Gerstner, W. (2017). Predicting non-linear dynamics by stable local learning in a recurrent spiking neural network. *Elife* 6, e28295

Glorot, X. and Bengio, Y. (2010). Understanding the difficulty of training deep feedforward neural networks. In *Proceedings of the thirteenth international conference on artificial intelligence and statistics* (JMLR Workshop and Conference Proceedings), 249–256

Gütig, R. (2016). Spiking neurons can discover predictive features by aggregate-label learning 351, aab4113. doi:10.1126/science.aab4113

Han, S., Yang, W., and Yuste, R. (2019). Two-color volumetric imaging of neuronal activity of cortical columns. *Cell reports* 27, 2229–2240

Hinton, G. et al. (2007). How to do backpropagation in a brain. In *Invited talk at the NIPS'2007 deep learning workshop*. vol. 656, 1–16

Illing, B., Gerstner, W., and Bellec, G. (2020). Towards truly local gradients with CLAPP: contrastive, local and predictive plasticity. *CoRR* abs/2010.08262

Illing, B., Gerstner, W., and Brea, J. (2019). Biologically plausible deep learning—but how far can we go with shallow networks? *Neural Networks* 118, 90–101

Inglebert, Y., Aljadeff, J., Brunel, N., and Debanne, D. (2020). Synaptic plasticity rules with physiological calcium levels. *Proceedings of the National Academy of Sciences* 117, 33639–33648. doi:10.1073/pnas.2013663117

Izhikevich, E. M. and Desai, N. S. (2003). Relating STDP to BCM 15, 1511–1523. doi:10.1162/089976603321891783

Keller, G. B. and Mrsic-Flogel, T. D. (2018). Predictive processing: a canonical cortical computation. *Neuron* 100, 424–435

Kempter, R., Gerstner, W., and van Hemmen, J. L. (1999). Hebbian learning and spiking neurons 59, 4498–4514. doi:10.1103/PhysRevE.59.4498

Kingma, D. P. and Ba, J. (2014). Adam: A method for stochastic optimization. *3rd International Conference on Learning Representations, ICLR 2015, San Diego, CA, USA, May 7-9, 2015, Conference Track Proceedings*

Koch, C. and Poggio, T. (1999). Predicting the visual world: silence is golden. *nature neuroscience* 2, 9–10

Kogo, N. and Trengove, C. (2015). Is predictive coding theory articulated enough to be testable? *Frontiers in computational neuroscience* , 111

Kriegeskorte, N. and Golan, T. (2019). Neural network models and deep learning. *Current Biology* 29, R231–R236

Lamsa, K., Heeroma, J., Somogyi, P., Rusakov, D., and Kullmann, D. (2007). Anti-hebbian long-term potentiation in the hippocampal feedback inhibitory circuit. *Science* 315, 1262–1266

Larkum, M. (2013). A cellular mechanism for cortical associations: an organizing principle for the cerebral cortex. *Trends in neurosciences* 36, 141–151

Larkum, M. E., Senn, W., and Lüscher, H.-R. (2004). Top-down dendritic input increases the gain of layer 5 pyramidal neurons. *Cerebral cortex* 14, 1059–1070

Larkum, M. E., Waters, J., Sakmann, B., and Helmchen, F. (2007). Dendritic spikes in apical dendrites of neocortical layer 2/3 pyramidal neurons. *Journal of Neuroscience* 27, 8999–9008

Larkum, M. E., Zhu, J. J., and Sakmann, B. (1999). A new cellular mechanism for coupling inputs arriving at different cortical layers. *Nature* 398, 338

Lazar, A., Pipa, G., and Triesch, J. (2009). Sorn: a self-organizing recurrent neural network. *Frontiers in computational neuroscience* , 23

LeCun, Y. (1998). The mnist database of handwritten digits. *http://yann. lecun. com/exdb/mnist/*

Liaw, R., Liang, E., Nishihara, R., Moritz, P., Gonzalez, J. E., and Stoica, I. (2018). Tune: A research platform for distributed model selection and training. *arXiv preprint arXiv:1807.05118*

Lillicrap, T. P., Santoro, A., Marris, L., Akerman, C. J., and Hinton, G. (2020). Backpropagation and the brain. *Nature Reviews Neuroscience* , 1–12

Lotter, W., Kreiman, G., and Cox, D. (2020). A neural network trained for prediction mimics diverse features of biological neurons and perception. *Nature machine intelligence* 2, 210–219

Luczak, A., McNaughton, B. L., and Kubo, Y. (2022). Neurons learn by predicting future activity. *Nature Machine Intelligence* 4, 62–72

Markram, H., Lübke, J., Frotscher, M., and Sakmann, B. (1997). Regulation of synaptic efficacy by coincidence of postsynaptic APs and EPSPs 275, 213–215. doi:10.1126/science.275.5297.213

Masquelier, T., Guyonneau, R., and Thorpe, S. J. (2009). Competitive stdp-based spike pattern learning. *Neural computation* 21, 1259–1276

Meulemans, A., Carzaniga, F., Suykens, J., Sacramento, J. a., and Grewe, B. F. (2020). A theoretical framework for target propagation. *Advances in Neural Information Processing Systems* 33, 20024–20036

Meulemans, A., Farinha, M. T., Cervera, M. R., Sacramento, J., and Grewe, B. F. (2022a). Minimizing control for credit assignment with strong feedback. In *International Conference on Machine Learning* (PMLR), 15458–15483

Meulemans, A., Farinha, M. T., Ordóñez, J. G., Aceituno, P. V., Sacramento, J., and Grewe, B. F. (2021a). Credit assignment in neural networks through deep feedback control. *arXiv preprint arXiv:2106.07887*

Meulemans, A., Farinha, M. T., Ordóñez, J. G., Aceituno, P. V., Sacramento, J., and Grewe, B. F. (2021b). Credit assignment in neural networks through deep feedback control. *CoRR* abs/2106.07887

[Dataset] Meulemans, A., Zucchet, N., Kobayashi, S., von Oswald, J., and Sacramento, J. (2022b). The least-control principle for learning at equilibrium. doi:10.48550/ARXIV.2207.01332

Mozafari, M., Ganjtabesh, M., Nowzari-Dalini, A., and Masquelier, T. (2019). Spyketorch: Efficient simulation of convolutional spiking neural networks with at most one spike per neuron. *Frontiers in neuroscience* , 625

Nishiyama, M., Hong, K., Mikoshiba, K., Poo, M.-m., and Kato, K. (2000). Calcium stores regulate the polarity and input specificity of synaptic modification 408, 584–588. doi:10.1038/35046067

Paszke, A., Gross, S., Massa, F., Lerer, A., Bradbury, J., Chanan, G., et al. (2019). Pytorch: An imperative style, high-performance deep learning library. In *Advances in Neural Information Processing Systems 32* (Curran Associates, Inc.). 8024–8035

Payeur, A., Guerguiev, J., Zenke, F., Richards, B., and Naud, R. (2021). Burst-dependent synaptic plasticity can coordinate learning in hierarchical circuits. *Nature neuroscience* 24, 1546

Pelgrom, M. J., Duinmaijer, A. C., and Welbers, A. P. (1989). Matching properties of mos transistors. *IEEE Journal of solid-state circuits* 24, 1433–1439

Rao, R. P. and Ballard, D. H. (1999). Predictive coding in the visual cortex: a functional interpretation of some extra-classical receptive-field effects. *Nature neuroscience* 2, 79–87

Rosenbaum, R. (2022). On the relationship between predictive coding and backpropagation. *Plos one* 17, e0266102

Sacramento, J., Costa, R. P., Bengio, Y., and Senn, W. (2018). Dendritic cortical microcircuits approximate the backpropagation algorithm. In *Advances in Neural Information Processing Systems 31*. 8721–8732

Saponati, M. and Vinck, M. (2021). Sequence anticipation and stdp emerge from a voltage-based predictive learning rule. *bioRxiv*

Saudargiene, A., Porr, B., and Wörgötter, F. (2004). How the shape of pre-and postsynaptic signals can influence stdp: a biophysical model. *Neural Computation* 16, 595–625

Scellier, B. and Bengio, Y. (2017). Equilibrium propagation: Bridging the gap between energy-based models and backpropagation. *Frontiers in computational neuroscience* 11, 24

Segal, M. (2018). Calcium stores regulate excitability in cultured rat hippocampal neurons 120, 2694–2705. doi:10.1152/jn.00447.2018

Sjöström, J., Gerstner, W., et al. (2010). Spike-timing dependent plasticity. *Scholarpedia* 35, 0–0

Sjöström, P. J., Turrigiano, G. G., and Nelson, S. B. (2001). Rate, timing, and cooperativity jointly determine cortical synaptic plasticity 32, 1149–1164. doi:10.1016/S0896-6273(01)00542-6

Song, S., Miller, K. D., and Abbott, L. F. (2000). Competitive hebbian learning through spike-timing-dependent synaptic plasticity 3, 919–926. doi:10.1038/78829

Toyoizumi, T., Pfister, J.-P., Aihara, K., and Gerstner, W. (2005). Generalized bienenstock–cooper–munro rule for spiking neurons that maximizes information transmission. *Proceedings of the National Academy of Sciences* 102, 5239–5244

[Dataset] Tschantz, A. (2020). A Python implementation of An Approximation of the Error Backpropagation Algorithm in a Predictive Coding Network with Local Hebbian Synaptic Plasticity

van Rossum, M. C. W., Bi, G. Q., and Turrigiano, G. G. (2000). Stable hebbian learning from spike timing-dependent plasticity 20, 8812–8821. doi:10.1523/JNEUROSCI.20-23-08812.2000

Vilimelis Aceituno, P., Ehsani, M., and Jost, J. (2020). Spiking time-dependent plasticity leads to efficient coding of predictions. *Biological cybernetics* 114, 43–61

Whittington, J. C. and Bogacz, R. (2017). An approximation of the error backpropagation algorithm in a predictive coding network with local hebbian synaptic plasticity. *Neural computation* 29, 1229–1262

Williams, L. E. and Holtmaat, A. (2019). Higher-order thalamocortical inputs gate synaptic long-term potentiation via disinhibition. *Neuron* 101, 91–102

Wittenberg, G. M. and Wang, S. S.-H. (2006). Malleability of spike-timing-dependent plasticity at the CA3-CA1 synapse 26, 6610–6617. doi:10.1523/JNEUROSCI.5388-05.2006

Xie, X. and Seung, H. S. (1999). Spike-based learning rules and stabilization of persistent neural activity. In *Advances in Neural Information Processing Systems*, eds. S. Solla, T. Leen, and K. Müller (MIT Press), vol. 12

Yang, S., Gao, T., Wang, J., Deng, B., Azghadi, M. R., Lei, T., et al. (2022a). Sam: a unified self-adaptive multicompartmental spiking neuron model for learning with working memory. *Frontiers in Neuroscience* 16

Yang, S., Linares-Barranco, B., and Chen, B. (2022b). Heterogeneous ensemble-based spike-driven few-shot online learning. *Frontiers in Neuroscience* 16

Yang, S., Tan, J., and Chen, B. (2022c). Robust spike-based continual meta-learning improved by restricted minimum error entropy criterion. *Entropy* 24, 455

Zappacosta, S., Mannella, F., Mirolli, M., and Baldassarre, G. (2018). General differential hebbian learning: Capturing temporal relations between events in neural networks and the brain. *PLoS computational biology* 14, e1006227

Zhang, S., Xu, M., Kamigaki, T., Do, J. P. H., Chang, W.-C., Jenvay, S., et al. (2014). Long-range and local circuits for top-down modulation of visual cortex processing. *Science* 345, 660–665. doi:10.1126/science.1254126
